# Organizational Behavior in Green Supply Chain Integration: Nexus Between Information Technology Capability, Green Innovation, and Organizational Performance

**DOI:** 10.3389/fpsyg.2022.874639

**Published:** 2022-03-22

**Authors:** Adnan Abbas, Xiaoguang Luo, Muhammad Umair Wattoo, Rui Hu

**Affiliations:** ^1^School of Economics and Management, Harbin University of Science and Technology, Harbin, China; ^2^School of Management, Jiangsu University, Zhenjiang, China; ^3^Management Sciences Department, The Islamia University of Bahawalpur, Bahawalpur, Pakistan

**Keywords:** green development, green supply chain, green innovation, performance, IT capability

## Abstract

Stakeholder pressure and public awareness of environmental protection drive organizations to improve environmental practices in the supply chain (SC), such as green supply chain integration (GSCI) and green innovation (GI). The use of information technology (IT) is crucial to manufacturing organizations’ GSCI and performance. However, the research on the relationship between IT capabilities, GSCI, GI and organizational performance is still limited. Therefore, empirical research is needed on the cognitive thinking of employees using IT capabilities to improve GSCI and organizational performance. The data for this study comes from SC personnel in manufacturing organizations through a structured questionnaires and was analyzed by employing structural equation modeling. Based on the results, this paper concludes that organizational IT capabilities positively affect the GSCI and improve organizational performance (environmental and operational performance). Furthermore, the study discovered that GI increases organizational performance and acts as a positive mediator in the link between GSCI and performance. The findings contribute to existing GSCI and GI knowledge, which can provide a bird’s eye-view to develop an organization’s IT capabilities to achieve competitive performance goals.

## Introduction

In the modern era, enterprises are active participants in the green production/green economy to achieve social and environmental performance (EP), not just financial performance (Abu Seman et al., 2019). Furthermore, the over-saturated GSC literature presents inconsistent advice and tactics that hamper the application of sustainable operations (Samad et al., 2021; McDougall et al., 2022). The GSCI is very important on GI, which is important to achieving operational performance (OP) and EP, not just the financial performance (Kong et al., 2021). GSCI has been construed by business and literature beyond a common theme of different definitions put forward by researchers through parallel approaches (Wu, 2013; Setyadi, 2019; Kong et al., 2020). Organizations may motivated in conducting green operations more efficiently to achieve greater performance (Hu et al., 2022) also by integrating with consumers and suppliers (Zhu et al., 2010). In the context of GSC, this integration may also be considered a vital organizational competency (Lo et al., 2018). Therefore, research requires a complete understanding of the relationship between GSCI and GI.

Furthermore, GSCI provides an opportunity and appropriate way for organizations to learn about the GSC. Previous GSCI research neglect the possible interaction between various forms of GSCI (Lo et al., 2018; Wong et al., 2020), while others fail to recognize the effect of technological advancement and organizational capabilities on GSCI and organizational performance (Wu, 2013; Al-Ghwayeen and Abdallah, 2018). Few researches has concentrated on the connection between GSC initiatives and organizational performance, e.g., (Green et al., 2012; Yu et al., 2014; Cousins et al., 2019; Sebastianelli and Tamimi, 2020). However, the consideration of these concepts is not clear enough, especially in developing countries where the concept of “go-green” is not mature enough. Meanwhile, the literature has found that leveraging capabilities within an organization can enhance organizational effectiveness, increase organizational value, reduce external pressure, and lead to better organizational performance (Zhu et al., 2010; Sebastianelli and Tamimi, 2020; Shahzad et al., 2020; Wang et al., 2020; Kitsis and Chen, 2021). Therefore, this study attempts to analyze the relationship between GSCI and organizational performance from the perspective of GI, combined with dynamic capability theory, so as to better explain this process.

Technological innovations are always accompanied by a degree of uncertainty about their outcomes, which can reduce their adoption and organizational performance (Hong et al., 2019). Therefore, in the current study context, it is essential to clarify how different capabilities manage the outcome uncertainty of GI in order to attain distinct performance advantages. According to Effendi et al. (2021), GI performance in GSC covers green process innovation and eco-friendly product innovation. Information processing theory offers a fresh look at the intricacies that underpin the various implications of GSCI dimensions and GI on organizational performance (Wong et al., 2020). Therefore, this research suggests and evaluates a comprehensive research model to analyze the possible impact of IT capabilities on GSCI that enhance organizational performance in the presence of GI.

Our work covers two major research concerns in order to add to the current literature. First, what effect do IT capabilities on GSCI have, thereby organizational performance? Second, how does GI mediate between GSCI and organizational performance? This study intends to give insights into the possible influence of IT capabilities on GSCI and its ramifications by answering these questions.

## Literature Review and Hypothesis Development

Organizations integrate SC to minimize operational expenses and increase customer service quality (Rao and Holt, 2005). SC integration promotes organizations to collaborate in order to build inter-organizational ties, merge business operations, and expedite knowledge transfer with business partners (Handfield et al., 2009; Yang and Tsai, 2019). According to Flynn et al. (2010), “the degree to which a manufacturer strategically collaborates with its SC partners and collaboratively manages intra- and inter-organization processes” is known as SCI. The concept of GSCI comes from green supply chain management (GSCM) and SCI literature (Wong et al., 2020), which integrate the environmental concern in the SCI. GSCI assesses how well a producer interacts with suppliers, customers, and internal departments to satisfy environmental criteria (Kong et al., 2021). GSCI aims to reduce environmental consequences, increase resource utilization, and promote long-term performance improvement by managing intra- and inter-organizational processes magnificently (Vachon and Mao, 2008; Cousins et al., 2019). GSCI is an essential source of technical innovation in enterprises, SC members (including suppliers and customers), and expertise and an important source of ideas (Effendi et al., 2021).

As proposed by SCI literature, there are three types of GSCI: green internal integration, green supplier integration, and green customer integration (Wu, 2013; Setyadi, 2019; Han and Huo, 2020). Green internal integration is when a producer conducts environmental management practices in an organization to achieve the intra-organizational process (Yu et al., 2014; Kong et al., 2020). Green external integration, which consists of both green supplier and green customer integration, reflects the extent to which manufacturing partners with its external partners handle inter-organizational green initiatives (Yu et al., 2014; Setyadi, 2019; Kong et al., 2021). Green supplier integration offers manufacturers the opportunity to learn about green practices employed by their key suppliers, such as design specifications for green products and eco-friendly operations (Lee et al., 2012). In contrast, green customer integration is conducive to distributing key market evidence, such as customer needs for green products, competitive information, etc. (Shafique et al., 2018; Kong et al., 2021).

Furthermore, GI, including product redesign, might be challenging to complete, but many firms choose GI that just modifies manufacturing and logistical procedures rather than redesigning goods (Bartlett and Trifilova, 2010). It is claimed that outlay in a green product or either in green process innovation may lead to significant differences in performance outcomes (Dangelico, 2016; Shen et al., 2020). So, we distribute GI into two phases one is green product innovation, and the other is green process innovation. Green product innovation comes when green ideas are incorporated into the (re)design of products and packaging in order to improve product quality and distinction (Handfield et al., 2005; Huang and Li, 2017). Green product design mostly includes major product technique and design (Wong et al., 2020).

In contrast, green process innovation is described as “the modifications during manufacturing processes and systems to ensure energy savings, pollution prevention, and waste recycling” (Li et al., 2016). Green process innovation involves decreasing waste and energy utilization while sourcing, construction, and logistics actions without redesigning the products (Wong et al., 2020). Other advantages of investing in green process innovation include organizations claiming environmental gains in logistics and supply chain activities, but customers may not realize environmental benefits with the same product design (Ma et al., 2017). Green product and process innovations, in theory, should lead to improved organizational performance. They do, however, necessitate different products and process methods, and their advantages are enjoyed in various sections of the GSC. Another significant finding is that analyzing how the integration processes employed in GSCI enrich the data analytics skills necessary for GI.

### Information Technology Capabilities

The role of IT in SCM has been emphasized in the past, for example, integrated information systems can improve the business performance of companies in the SC (Thöni and Tjoa, 2017). In the information science literature, IT capabilities are described as the specified capability to acquire, implement, and use IT resources to assist firms to achieve a competitive edge (Wang et al., 2012; Soltany et al., 2018). Scholars gradually view IT capabilities as lower-order capabilities that enable them to develop higher-order capabilities such as responsiveness, new product development dynamics, and operations (Liu et al., 2013; Wong et al., 2020). IT capabilities ensure that the business can handle the wealth of information and knowledge involved in SC practices to benefit from these practices (Wei et al., 2020). However, several studies have shown that IT may aid in the advancement of SCM capabilities (Wu et al., 2006; Shahzad et al., 2020). Thus, we incorporate four core IT capabilities: IT-management “organizational capabilities related to IT infrastructure, costs, staff development, etc.,” IT-development “an organization’s capability to accurately meet its business requirements through IT system development and implementation,” IT-intensity “organizational capabilities related to the practical use of IT to achieve competitive advantages” and IT-Assimilation “capability to diffuse and routinize IT applications in business process” (Ravichandran and Lertwongsatien, 2005; Liang et al., 2007; Liu et al., 2013; Shahzad et al., 2020). Building on previous contributions of IT in SC research, this study measures four categories of organizational IT capabilities that influence the development of a robust GSCI system. Thus, we posit that:

**H1a:** IT management capability positively influence GSCI.

**H1b:** IT development capability positively influences GSCI.

**H1c:** IT intensity positively influence GSCI.

**H1d:** IT assimilation positively influence GSCI.

### Green Supply Chain Integration, Green Innovation, and Organizational Performance

GSCI emphasizes launching a GSCM strategy that comprehensively considers the environmental impact and resource utilization and emphasizes green cooperation to achieve valuable sustainable development goals (Han and Huo, 2020). The integration of green suppliers and customers can be regarded as green external integration. Green external integration refers to the degree of environmental cooperation between manufacturers and external partners, such as common sense of environmental responsibilities, joint problem solving, and joint realization of environmental goals (Kong et al., 2020). Setting common environmental goals with suppliers, reassuring suppliers to use advanced technologies in the manufacturing process, imposing specific environmental requirements on supplier product design, and jointly developing an environmentally friendly new product are all examples of green supplier integration (Wu, 2013; Kong et al., 2021). While, green customer integration practices to attain environmental goals, jointly deciding on eco-strategic options to reduce the environmental impact of new products (Wu, 2013; Kong et al., 2021), and performing collaborative planning to realize customer requirements and meet environmental safety needs (Han and Huo, 2020).

On the other hand, green internal integration is concerned with cooperative environmental initiatives within organizations (Ni and Sun, 2019). Internal integration acknowledges the need to closely coordinate diverse divisions within a company and remove obstacles to communication and collaboration across departments (Flynn et al., 2010). Internal integration allows design, production, and marketing to collaborate easily in order to facilitate concurrent engineering and optimize product and process improvements (Wu, 2013). As a result, SC partners may participate in environmental initiatives, contribute crucial competencies for GI, stimulate cooperation, and build cross-company problem-solving methods (Wong et al., 2012; Cai et al., 2020).

Research suggests that there may be differences in the performance advantages of green product and process innovations. Green product and process innovations are often correlated with a competitive edge and organizational performance (Chen et al., 2006). Green process innovation has been demonstrated to be inconsequential in competitive and economic advantages. It is difficult to explain this mixed effect (Wong et al., 2020). It may significantly impact organizational performance and play a mediating role in the relationship between GSCI and organizational performance. Given the above considerations, the following assumptions are made:

**H2a:** GSCI positively influences GI.

**H2b:** GSCI positively influences OP.

**H2c:** GSCI positively influences EP.

### Green Innovation and Organizational Performance

Innovation is a critical technique for improving organizations’ capacity to sustain a competitive edge (Huang and Li, 2017). GI strategy comes from green organizational performance, including environmental support behavior and norms. Corporate managers with environmental protection culture are more expected to execute environmental protection strategies and increase organizational GI (Sdrolia and Zarotiadis, 2019). Therefore, organizations differentiate their GI capabilities by adjusting their organizational culture to strengthen environmental quality standards (Weng et al., 2015; Gupta and Barua, 2018). With the ongoing degradation of the natural environment, enterprises face pressure from different stakeholders (internal or external) to diminish the adverse effect of products on the environment (Hu et al., 2022). GI in products and processes decreases environmental effects and boosts a competitive performance (Xie et al., 2016; Zhang et al., 2019). Green product innovation empowers organizations to react to market and government environmental demands, boost resource efficiency and optimize environmental advantages throughout the product life cycle (Dangelico, 2016; Wong et al., 2020). To comply with environmental requirements, enterprises must cut clean production costs and pollutant emissions through green process innovation (Xie et al., 2016; Wong et al., 2020).

Organizations increasingly use GI to minimize hazardous waste emissions and adapt to external pressure (Zhang et al., 2020). However, GI effectively requires resources across organizational/functional boundaries. The study of Wang (2019) found a partial mediating impact of GI between green organizational culture and green performance. Organizations should collaborate with SC partners to turn to the GSCI paradigm (Cai et al., 2020). While prior research has shown a link between integration and creativity, GSCI gives chances to acquire and deploy resources (Wu, 2013; Dangelico, 2016; Effendi et al., 2021). Research also supports that environmental management practices, directly and indirectly, affect EP (Thong and Wong, 2018; Xu et al., 2020). The prior studies also proved that the customers demand high levels of OP and EP from their suppliers, and the organizations have a similar attitude toward their stakeholders (Fawcett et al., 2007; Gholami et al., 2013; Yu et al., 2014). Therefore, in the current study context, the author explores GI’s direct and indirect impact on organizational performance (OP and EP).

**H3a:** GI positively influences OP.

**H3b:** GI mediates the relationship between GSCI and OP.

**H4a:** GI positively influences EP.

**H4b:** GI mediates the relationship between GSCI and EP.

**H5:** OP positively influences EP.

**FIGURE 1:**
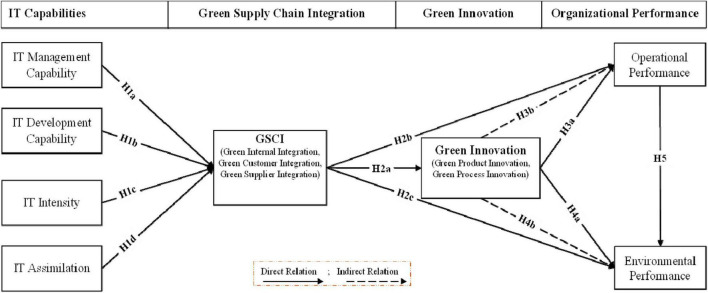
Proposed model.

Figure 1 represents the proposed research framework.

## Materials and Methods

### Measurement of Variables

To realistically guarantee the content validity of the research model, this study prepared a structured questionnaire and constructed and operationalized the items of all exogenous variables. The survey items were designed to induce agreement or disagreement among respondents based on a 7-point Likert scale, including a series of statements about seven key drivers, ranging from “strongly disagree = 1” to “strongly agree = 7.” Items are taken from previous studies and set according to the context. Before data collection, a group of research scholars revised and discussed the face validity of the questionnaire.

### Data Collection and Samples

The research design used quantitative methods and a questionnaire survey. The required data collection work is carried out in different manufacturing organizations in Pakistan. Previous studies have proven that manufacturing is one of the leading causes of environmental degradation (Jantunen et al., 2005; Green et al., 2012; Samad et al., 2021). Therefore, we aim to identify organizational development behaviors in GSCI that can improve the operational and environmental performance of manufacturing organizations. SC managers from Pakistani manufacturing organizations were chosen as the target sample to study organizational behavior regarding the GSCI. The data for the study was gathered via online questionnaires. The authors reached top management of the manufacturing organizations via email and phone calls. We discussed the purpose with them and sent them a link to the questionnaire upon approval. In addition, because of the hometown, the authors personally visited the industrial centers of various Pakistani cities and were visited to different manufacturing organizations. The authors initiated the data collection process in August 2021; using all personal and professional resources, we got 421 responses in December 2021. A combination of strategies to improve response rates, including follow-up calls and in-person visits to the organization, helped us achieve the desired response. The sample size was adequate to test the research model in structural equation modeling as described by prior scholars (Hair et al., 2012). From the screening of collected data, we excluded 18 unengaged responses. The final 403 responses were included in the data analysis.

### Demographics of Respondents

Table 1 recaps the characteristics (gender, experience, and income level) of the main participants of this study. Men accounted for 65.1% of the respondents, and women accounted for 35%. The results show that 19.6% of the respondents have 1–3 years of work experience in their institutions, 44.4% have 4–6 years of work experience, and 36% have more than 6 years of work experience. Most of the respondents belonged to the high-income group: only 14.4% of people had incomes below 50,000 (Pakistan rupee), 37% of people belonged to 50,000–1 lac, and the remaining 48.6% belonged to income groups higher than 1 lac per month.

**TABLE 1:** Respondent’s profile.

Category		Frequency	%age
Gender	Male	262	65.0
	Female	141	35.0
	Total	403	100.0
Experience	1–3 years	79	19.6
	4–6 years	179	44.4
	7–9 years	81	20.1
	Above 10 years	64	15.9
	Total	403	100.0
Monthly income (in Pakistani rupee)	Less than 50,000	58	14.4
	50,000–100,000	149	37.0
	100,000–150,000	125	31.0
	Above 150,000	71	17.6
	Total	403	100.0

## Results

### Measurement Model

#### Reliability and Convergent Validity

This study used Smart-PLS v3 software to perform confirmatory factor analysis (CFA) to confirm the convergence validity of each item. Table 2 demonstrates the value of this study’s reliability and convergence validity analysis. The Cronbach’s alpha values of all components are between 0.878 and 0.944, higher than the threshold. The composite reliability (CR) is 0.923–0.953, and the average variance extracted (AVE) is 0.710–0.827. The recommended value of Cronbach’s alpha and CR should be greater than 0.7, and the recommended value of AVE should be greater than 0.5, indicating that the instrument is effective and reliable (Fornell and Larcker, 1981b; Hair et al., 2014). Therefore, the results depicted in this article show no problem with the convergence validity and reliability of the data used in this study.

**TABLE 2:** Convergent validity and reliability.

Constructs	Cronbach’s alpha	rho_A	CR	AVE
EP	0.916	0.917	0.937	0.750
GCI	0.892	0.897	0.933	0.823
GII	0.929	0.932	0.947	0.781
GPCI	0.930	0.930	0.950	0.826
GPDI	0.888	0.889	0.923	0.749
GSI	0.897	0.898	0.924	0.710
ITA	0.878	0.897	0.925	0.805
ITDC	0.925	0.926	0.947	0.817
ITI	0.895	0.895	0.935	0.827
ITMC	0.905	0.916	0.934	0.779
OP	0.944	0.948	0.953	0.717

#### Discriminant Validity

The examination of discriminant validity has to turn into a widely recognized concept for evaluating the connection between prospective components (Hair et al., 2014). Three strategies were employed in this study to measure discriminant validity; first, by linking the correlation of each factor to the square root of the AVE; second, to determine the significance of the survey items, we use item loadings and cross-loadings; third, using the Heterotrait—Monotrait (HTMT) ratio (Fornell and Larcker, 1981b; Hair et al., 2014; Henseler et al., 2014).

The association between factors and AVE was coupled to test the instrument’s validity—called the Fornel-Larcker standard. In Table 3, diagonal values show that the AVE square root is greater than the values of inter-construct correlation. This demonstrates no discriminant validity problem (Fornell and Larcker, 1981a).

**TABLE 3:** Fornell-Larcker criterion.

	EP	GCI	GII	GPCI	GPDI	GSI	ITA	ITDC	ITI	ITMC	OP
EP	**0.866**										
GCI	0.514	**0.907**									
GII	0.493	0.458	**0.884**								
GPCI	0.523	0.486	0.478	**0.909**							
GPDI	0.502	0.461	0.520	0.625	**0.865**						
GSI	0.478	0.467	0.518	0.461	0.451	**0.843**					
ITA	0.268	0.333	0.399	0.342	0.348	0.371	**0.897**				
ITDC	0.527	0.455	0.477	0.530	0.553	0.429	0.369	**0.904**			
ITI	0.466	0.455	0.473	0.494	0.510	0.418	0.367	0.479	**0.909**		
ITMC	0.326	0.393	0.321	0.311	0.379	0.398	0.318	0.354	0.432	**0.883**	
OP	0.491	0.455	0.486	0.489	0.524	0.445	0.330	0.453	0.488	0.373	**0.847**

*“Diagonal and Bold-faced values are the square root of the average variance extracted from each construct.” p < 0.05.*

Prior research has explored using cross-loading criteria to assess discriminant validity (Fornell and Larcker, 1981b; Liu et al., 2016a; Shahzad et al., 2020). The item loadings and cross-loadings for all corresponding values are shown in Table 4, suggesting that the item loading of each factor is larger than the cross-loading of other possible factors. This indicates that the validity of the discrepancy is adequate by meeting the cross-loading criteria.

**TABLE 4:** Constructs cross-loadings.

	EP	GCI	GII	GPCI	GPDI	GSI	ITA	ITDC	ITI	ITMC	OP
EP1	**0.854**	0.425	0.430	0.452	0.431	0.386	0.229	0.478	0.384	0.265	0.416
EP2	**0.864**	0.450	0.426	0.467	0.420	0.410	0.239	0.465	0.404	0.265	0.392
EP3	**0.870**	0.396	0.398	0.438	0.419	0.425	0.162	0.438	0.397	0.270	0.442
EP4	**0.905**	0.418	0.434	0.451	0.424	0.412	0.218	0.446	0.383	0.266	0.434
EP5	**0.835**	0.527	0.444	0.452	0.476	0.434	0.305	0.455	0.446	0.340	0.438
GCI1	0.452	**0.884**	0.370	0.456	0.424	0.386	0.268	0.391	0.377	0.297	0.393
GCI2	0.429	**0.906**	0.421	0.416	0.376	0.415	0.303	0.397	0.367	0.365	0.404
GCI3	0.514	**0.931**	0.452	0.450	0.454	0.466	0.333	0.448	0.488	0.401	0.440
GII1	0.429	0.377	**0.855**	0.409	0.472	0.424	0.310	0.443	0.433	0.295	0.438
GII2	0.447	0.443	**0.907**	0.415	0.479	0.492	0.348	0.446	0.423	0.265	0.461
GII3	0.450	0.431	**0.921**	0.421	0.464	0.459	0.392	0.398	0.421	0.291	0.439
GII4	0.412	0.325	**0.842**	0.411	0.407	0.416	0.321	0.396	0.353	0.248	0.385
GII5	0.441	0.439	**0.890**	0.455	0.473	0.490	0.386	0.426	0.456	0.318	0.420
GPCI1	0.500	0.472	0.487	**0.899**	0.636	0.468	0.335	0.520	0.527	0.327	0.505
GPCI2	0.449	0.445	0.415	**0.913**	0.539	0.402	0.278	0.486	0.429	0.275	0.419
GPCI3	0.464	0.416	0.401	**0.909**	0.523	0.400	0.307	0.462	0.402	0.241	0.397
GPCI4	0.485	0.431	0.431	**0.914**	0.569	0.402	0.323	0.458	0.434	0.285	0.451
GPDI1	0.474	0.428	0.458	0.559	**0.877**	0.387	0.295	0.539	0.447	0.363	0.418
GPDI2	0.389	0.376	0.423	0.542	**0.839**	0.332	0.291	0.437	0.386	0.233	0.459
GPDI3	0.493	0.426	0.465	0.552	**0.898**	0.452	0.304	0.475	0.487	0.333	0.471
GPDI4	0.377	0.362	0.453	0.508	**0.846**	0.387	0.316	0.462	0.444	0.383	0.468
GSI1	0.452	0.451	0.504	0.434	0.471	**0.834**	0.327	0.421	0.407	0.319	0.424
GSI2	0.384	0.431	0.436	0.398	0.335	**0.808**	0.349	0.367	0.342	0.320	0.349
GSI3	0.362	0.331	0.384	0.306	0.335	**0.882**	0.293	0.283	0.300	0.325	0.328
GSI4	0.396	0.370	0.417	0.337	0.359	**0.894**	0.325	0.355	0.354	0.386	0.370
GSI5	0.413	0.377	0.431	0.461	0.390	**0.792**	0.266	0.372	0.348	0.328	0.397
ITA1	0.293	0.353	0.378	0.373	0.367	0.388	**0.946**	0.378	0.352	0.302	0.334
ITA2	0.220	0.298	0.376	0.287	0.336	0.313	**0.916**	0.345	0.343	0.333	0.287
ITA3	0.201	0.235	0.317	0.250	0.219	0.291	**0.825**	0.260	0.289	0.213	0.262
ITDC1	0.454	0.413	0.427	0.485	0.507	0.344	0.340	**0.890**	0.410	0.326	0.401
ITDC2	0.465	0.385	0.431	0.463	0.473	0.419	0.322	**0.886**	0.446	0.287	0.395
ITDC3	0.508	0.426	0.441	0.476	0.518	0.423	0.335	**0.911**	0.467	0.336	0.413
ITDC4	0.477	0.422	0.426	0.495	0.502	0.359	0.339	**0.930**	0.405	0.331	0.427
ITI1	0.420	0.433	0.402	0.463	0.411	0.413	0.345	0.423	**0.878**	0.342	0.440
ITI2	0.424	0.402	0.442	0.418	0.462	0.336	0.293	0.472	**0.941**	0.398	0.446
ITI3	0.427	0.405	0.445	0.465	0.518	0.387	0.360	0.411	**0.907**	0.438	0.444
ITMC1	0.294	0.380	0.275	0.297	0.349	0.401	0.309	0.352	0.407	**0.899**	0.332
ITMC2	0.316	0.360	0.265	0.319	0.361	0.302	0.264	0.375	0.370	**0.884**	0.334
ITMC3	0.308	0.357	0.320	0.275	0.341	0.405	0.313	0.301	0.411	**0.911**	0.380
ITMC4	0.226	0.281	0.268	0.201	0.284	0.278	0.225	0.214	0.328	**0.834**	0.259
OP1	0.403	0.339	0.375	0.398	0.427	0.380	0.285	0.355	0.365	0.289	**0.850**
OP2	0.445	0.418	0.438	0.429	0.455	0.348	0.265	0.415	0.420	0.254	**0.885**
OP3	0.412	0.377	0.456	0.418	0.499	0.420	0.244	0.417	0.459	0.388	**0.859**
OP4	0.412	0.395	0.424	0.407	0.437	0.382	0.299	0.330	0.430	0.291	**0.847**
OP5	0.296	0.279	0.266	0.312	0.342	0.299	0.223	0.263	0.338	0.256	**0.790**
OP6	0.448	0.380	0.459	0.464	0.481	0.423	0.307	0.462	0.461	0.418	**0.820**
OP7	0.470	0.419	0.461	0.462	0.463	0.407	0.314	0.429	0.396	0.265	**0.857**
OP8	0.402	0.453	0.359	0.388	0.416	0.330	0.284	0.350	0.417	0.352	**0.863**

*“All factor loadings are significant at the p < 0.001 level.” Bold values are the item loadings.*

Finally, the HTMT ratio near to one is suggesting that the path analysis lacks discriminatant validity (Fornell and Larcker, 1981b). We also use the HTMT ratio; as shown in Table 5, the highest value is 0.686, which is less than the criterion (Henseler et al., 2014, 2016), confirming discriminant validity adequacy.

**TABLE 5:** HTMT ratio criterion.

	EP	GCI	GII	GPCI	GPDI	GSI	ITA	ITDC	ITI	ITMC
GCI	0.565									
GII	0.534	0.499								
GPCI	0.565	0.533	0.513							
GPDI	0.554	0.517	0.572	0.686						
GSI	0.525	0.519	0.564	0.503	0.502					
ITA	0.294	0.371	0.440	0.374	0.389	0.414				
ITDC	0.572	0.500	0.515	0.571	0.610	0.467	0.406			
ITI	0.514	0.506	0.518	0.540	0.572	0.463	0.412	0.525		
ITMC	0.355	0.432	0.348	0.336	0.422	0.436	0.350	0.385	0.477	
OP	0.521	0.491	0.510	0.515	0.569	0.478	0.358	0.477	0.527	0.397

### Path Model

**FIGURE 2:**
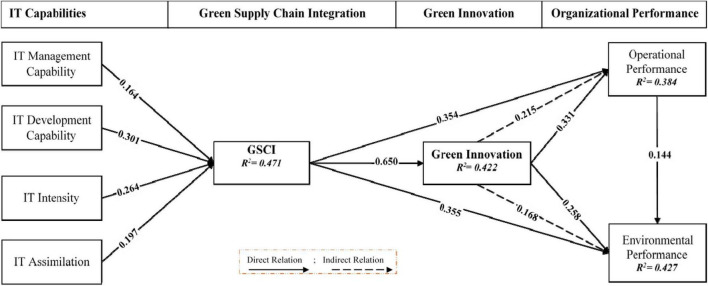
SEM results for hypotheses testing.

After investigating the research model’s reliability, convergence validity, and discriminant validity, a path model was employed to calculate the proposed linkages between factors (Hair et al., 2014; Henseler et al., 2016). Results are in Figure 2; all exogenous constructs in this investigation are strongly and positively linked with endogenous structures. Table 6 represent SEM results with the Bootstrapping path analysis. It indicates that the value of t-statistic is greater than the threshold value of 1.96, which proves that the relationship between quasi-variables is significant (Streukens and Leroi-Werelds, 2016; Hair et al., 2017). The *p*-value is also given to determine the significance. The R-squared value of the EP was 0.427, indicating that these selected variables represent a 42.7% variation. Similarly, the R-square of the adjusted OP is 0.384, indicating that changes in GSCI and GI account for 38.4%. The R-squared value of GI is 0.422, and the R-squared value of GSCI is 0.471, indicative of the actual contribution of the chosen factors.

**TABLE 6:** SEM results.

Hypotheses	Original sample (O)	Sample mean (M)	S. D	T Statistics (|O/STDEV|)	*P*-Values
H1a = ITMC - > GSCI	0.164	0.164	0.038	4.332	0.000
H1b = ITDC - > GSCI	0.301	0.302	0.038	7.834	0.000
H1c = ITI - > GSCI	0.264	0.262	0.044	6.011	0.000
H1d = ITA - > GSCI	0.197	0.197	0.047	4.212	0.000
H2a = GSCI - > GI	0.650	0.649	0.042	15.522	0.000
H2b = GSCI - > OP	0.354	0.354	0.043	8.219	0.000
H2c = GSCI - > EP	0.355	0.354	0.060	5.938	0.000
H3a = GI - > OP	0.331	0.330	0.047	6.975	0.000
H3b = GSCI - > GI - > OP	0.215	0.214	0.036	5.928	0.000
H4a = GI - > EP	0.258	0.257	0.055	4.689	0.000
H4b = GSCI - > GI - > EP	0.168	0.167	0.038	4.385	0.000
H5 = OP - > EP	0.144	0.144	0.050	2.913	0.004

In Table 6, SEM outcomes prove that the coefficient between ITMC and GSCI is 0.164. The results show that ITMC has a positive contribution to the GSCI. The beta coefficient between ITDC and GSCI is *b* = 0.301. The beta coefficients of ITI and ITA are *b* = 0.264 and 0.197. These outcomes disclose that IT capabilities have a significant and positive impact on GSCI, a second-order structural measurement. The research results show that ITMC, ITDC, ITI, and ITA play an essential role in improving the organization’s GSCI. Based on these empirical findings, H1a, H1b, H1c, and H1d are statistically supported.

In addition, the beta coefficient of GSCI is 0.650, implying that it has a significant effect on GI. So, accept H2a. GSCI is positively correlated with OP and EP of the organization. The beta coefficient value indicates a significant positive correlation between GSCI and OP (0.354) and between GSCI and EP (0.355). Thus, H2b and H2c are acceptable. The findings also demonstrate that GI has a considerable influence on the organization’s OP and EP. Table 6 explains the beta value at 0.331 and 0.258 of GI and EP. This suggests that IT capabilities substantially impact the organization’s inclusive performance. So, we also accept H3a and H4a.

This study also assumes that GI plays a mediating (or indirect) role in the relationship between GSCI and organizational performance. The outcomes are displayed in Figure 2 and Table 6. The indirect GI value between the relationship of GSCI and OP is (0.215), while the GI beta of the link between GSCI and EP is (0.168). Therefore, H3b and H4b were accepted. Finally, the research results show that the higher the OP, the higher the EP. OP is significantly positively correlated with beta value b of EP coefficient 0.144. So, accept H5.

### Common Method Bias

CMB is necessary for researchers to conduct research using independent and dependent constructs obtained from the same questionnaire tool. It was determined by single-factor analysis using the Harman test, which stipulated that a single factor should account for no more than 50% of the total variance (Morrison and Harman, 1961; Podsakoff et al., 2012). Our tests showed that a single factor accounted for 38%. At the same time, the inner variance inflation factor (VIF) was also employed to check the CMB. We found that these values ranged from 1.330 to 1.938; Thus, CMB is not a concern of this study.

## Discussion and Conclusion

The growing trend toward eco-modernization promotes the organizational capabilities to conduct their business in an environmentally friendly manner among their partners. This study establishes a conceptual model to examine the relationship between GSCI, GI, IT capabilities, and performance. The results showed that the development and implementation of IT capabilities increased the GSCI of SC partners and had a positive impact that motivated organizations to use GI practices to achieve performance goals. The findings extended the existing theory of dynamic capability in the SC literature. Consideration of various dimensions of IT capabilities as organizational dynamic capability creates a relevant understanding of the dynamic capability view. Dynamic capabilities represent a strategic routine for organizations to implement new resource allocation to adapt to rapidly changing environments (Teece, 2007; Huang and Li, 2017). Businesses can develop IT capabilities and modify their SC procedures to meet the challenges of GI through GSCI. This study empirically proved that IT capabilities have a positive impact on the GSCI.

Furthermore, our results highlight the importance of coordination in GSCI, GI, and organizational performance. Studies also proved that proactive GI can prepare organization for environmental risk management and sustainable environmental improvement capabilities (Huang and Li, 2017). External integration is comprised of two major components: supplier and customer integration, which promote knowledge sharing, cooperative development, and environmental engagement across SC partners. Internal integration also equally important to support the consistency of SC activities within the organization (Wu, 2013; Kong et al., 2020; Wong et al., 2020). The results of this study are also consistent with he prior studies.

This study also highlights the mediating role of enterprise GI in the relationship between GSCI and organizational performance. Green organization integration can help SC partners achieve their corporate social responsibility and sustainable development through the implementation of GI practices. Furthermore, this research reported a strong link between GSCI and organizational performance (OP and EP). This study emphasizes the significance of GSCI for Pakistani manufacturing organizations seeking to improve their competitiveness, operational and environmental performance. The positive, strong, and direct effects of GSCI on the GI show that GSCI is important for improving the GI; this is also consistent with recent studies (Wu, 2013; Wong et al., 2020; Junaid et al., 2022). It also illustrates the significance of implementing GI methods in industrial sector, which represents a new strategic approach for managers.

## Implications and Future Directions

### Implications

This research’s findings have the following academic and managerial contributions. First, we have identified the impact of IT capabilities on GSCI. The results help complement SC theory, especially in the GSCM, by examining the impact of IT capabilities that an organization uses to improve its GSCI. The outcomes support the conclusion of prior research that integrated IT in SC leads to higher SCI levels (Wu, 2013; Prajogo et al., 2016; Kim, 2017) and expand the range of available research in GSCI. Second, this paper expands the existing knowledge base of GSCI and GI in developing countries like Pakistan, where the GSCM concept has not attained its maturity. Third, this article highlights the prospective of GSCI and GI for improving operational and environmental performance that should be investigated further. It also develops a tried-and-true conceptual paradigm that aids and simplifies the deployment of IT capabilities in GSCI and GI practices by local SC partners. Furthermore, the models generated may assist manufacturers in identifying the difference between their present and desired practices compared to their rivals and design the appropriate strategies to close this gap.

This study also gives practical guidance for SC managers to use IT capabilities to achieve green development goals effectively. Today’s SC managers know that IT is essential but often do not thrive in using IT effectively (Su et al., 2021), not because companies have these IT systems that they are superior to, but there are also many failures in this area. The results revealed a positive correlation between IT capability and GSCI, and GSCI significantly impacts organizational performance. This has pointed out the direction and order for information system construction in GSCM. When organizations intend to use IT for GSCM, they first need to build and enrich IT capabilities within the focus organization. Subsequently, they must seek information integration with SC partners to improve the GSCI. Similarly, the study findings can assist manufacturers in identifying key IT capabilities and other indicators for successful implementation of GSCI and effectively implementing strategies to enhance possible GI practices leading to organizational performance.

### Limitations and Future Directions

We mentioned several limitations, and future research aims to address these limitations. Firstly, this study only takes green product innovation and green process innovation as the GI, with certain limitations. To further extend the understanding of GI’s role in the development of organizational performance, future researchers can add a variety of other factors, such as green management innovation and green marketing innovation, into future research. Secondly, because this study adopts a cross-sectional survey, the results limit the implementation to some extent, and a longitudinal survey can lead to a further intensive examination. Finally, the study is based on the employees’ perceptions. Although subjective assessments based on respondents’ impressions are significantly associated with objective measurements, more reliability is projected if secondary data is employed to gauge organizational performance in future research.

## Data Availability Statement

The raw data supporting the conclusions of this article will be made available by the authors, without undue reservation.

## Ethics Statement

The studies involving human participants were reviewed and approved by the Department of Management Science, The Islamia University of Bahawalpur, Pakistan. Written informed consent for participation was not required for this study in accordance with the national legislation and the institutional requirements.

## Author Contributions

XL: supervision, fund acquisition, project administration, and visualization. AA: conceptualization, writing—original draft preparation, methodology, software, and formal analysis. MW: data Collection, writing—review and Editing, and validation. RH: software, formal analysis, and writing—review and Editing. All authors contributed to the article and approved the submitted version.

## Conflict of Interest

The authors declare that the research was conducted in the absence of any commercial or financial relationships that could be construed as a potential conflict of interest.

## Publisher’s Note

All claims expressed in this article are solely those of the authors and do not necessarily represent those of their affiliated organizations, or those of the publisher, the editors and the reviewers. Any product that may be evaluated in this article, or claim that may be made by its manufacturer, is not guaranteed or endorsed by the publisher.
