# Corrigendum: Organizational Behavior in Green Supply Chain Integration: Nexus Between Information Technology Capability, Green Innovation, and Organizational Performance

**DOI:** 10.3389/fpsyg.2022.986142

**Published:** 2022-07-25

**Authors:** Adnan Abbas, Xiaoguang Luo, Muhammad Umair Wattoo, Rui Hu

**Affiliations:** ^1^School of Economics and Management, Harbin University of Science and Technology, Harbin, China; ^2^School of Management, Jiangsu University, Zhenjiang, China; ^3^Management Sciences Department, The Islamia University of Bahawalpur, Bahawalpur, Pakistan

**Keywords:** green development, green supply chain, green innovation, performance, IT capability

In the published article, there was an error in the author list. Author “Xiaoguang Luo” was erroneously listed in the wrong position. The corrected author list appears below.

“Adnan Abbas, Xiaoguang Luo, Muhammad Umair Wattoo and Rui Hu.”

The authors apologize for this error and state that this does not change the scientific conclusions of the article in any way. The original article has been updated.

## Publisher's note

All claims expressed in this article are solely those of the authors and do not necessarily represent those of their affiliated organizations, or those of the publisher, the editors and the reviewers. Any product that may be evaluated in this article, or claim that may be made by its manufacturer, is not guaranteed or endorsed by the publisher.

