# Supplementary material for: Organizational Behavior in Green Supply Chain Integration: Nexus Between Information Technology Capability, Green Innovation, and Organizational Performance
Source: Front Psychol. 2022 Mar 22;13:874639. doi: 10.3389/fpsyg.2022.874639 (PMC8982776; doi:10.3389/fpsyg.2022.874639)
Supplement: Supplementary file 1 [file Data_Sheet_1.docx]

**Appendix:**

**Survey Items**

**IT Management Capability** (Shahzad et al., 2020)

ITMC1: “Our IT function is good at managing contracts with software vendors”

ITMC2: “Our IT function can effectively manage IT assets”

ITMC3: “Our IT function can maintain an efficient budget for IT operations”

ITMC4: “Our IT function can satisfy business requirements timely”

**IT Development Capability** (Shahzad et al., 2020)

ITDC1: “Our organization can purchase suitable information systems to meet business needs”

ITDC2: “Our organization can implement the information systems that meet business needs”

ITDC3: “Our organization can develop information systems that meet business needs”

ITDC4: “Our organization has strong IT project management skills”

**IT Intensity** (Shahzad et al., 2020)

ITI1: “IT is used extensively by our competitors”

ITI2: “IT is used extensively by our suppliers and business partners”

ITI3: “IT is a critical means to interact with customers in this industry”

**IT Assimilation** (Liu et al., 2016b)

ITA1: “Our organization can implement IT in many business processes”

ITA2: “Our organization can implement IT in a large number of functional areas”

ITA3: “The extent to which IT is used in our organization (e.g., operation, management, and decision making) is high”

**Green Internal Integration** (Kong et al., 2020)

GII1: “Senior and middle managers are committed to GSCM in achieving environmental goals”

GII2: “Our organization is developing a mutual understanding of responsibilities regarding environmental performance”

GII3: “We are working together to reduce environmental impact of our activities”

GII4: “We are conducting joint planning to anticipate and resolve environmental-related problems”

GII5: “We are making joint decisions about ways to reduce the environmental impact of our products”

**Green Customer Integration** (Kong et al., 2020)

GCI1: “Our organization is achieving environmental goals through joint planning”

GCI2: “Our organization is cooperating with customers to reduce environmental impact of our products”

GCI3: “Our organization is cooperating with customers for cleaner production, green packaging, or other environmental activities”

**Green Supplier Integration** (Kong et al., 2020)

GSI1: “Our organization is collaborating with suppliers to set up environmental goals”

GSI2: “Our organization is implementing environmental audit for suppliers’ internal management”

GSI3: “Our organization is providing suppliers with environmental design requirements related to design specifications and cleaner production technology”

GSI4: “Our organization is requiring suppliers to implement environmental management or obtain third-party certification of environmental management system (e.g., ISO 14001)”

GSI5: “Our organization is selecting suppliers according to environmental criteria”

**Green Product Innovation** (Kong et al., 2020)

GPDI1: “Our organization chooses the materials of the product that produce the least amount of pollution for conducting the product development or design”

GPDI2: “Our organization chooses the materials of the product that consume the least amount of energy and resources for conducting the product development or design”

GPDI3: “Our organization uses the fewest number of materials to comprise the product for conducting the product development or design”

GPDI4: “Our organization would circumspectly deliberate whether the product is easy to recycle, reuse, and decompose for conducting the product development or design”

**Green Process Innovation** (Kong et al., 2020)

GPCI1: “The manufacturing process of our firm effectively reduces the emission of hazardous substances or waste”

GPCI2: “The manufacturing process of our firm recycles waste and emission that allow them to be treated and reused”

GPCI3: “The manufacturing process of our firm reduces the consumption of water, electricity, coal, or oil”

GPCI4: “The manufacturing process of our firm reduces the use of raw materials”

**Operational performance** (Fawcett et al., 2007; Shahzad et al., 2020)

OP1: “Our organization’s effectiveness in fulfilling requirements”

OP2: “Our organization’s effectiveness in responding to changes in market demand”

OP3: “Our organization’s effectiveness in on-time delivery”

OP4: “Reduction in lead time to fulfill customers’ orders”

OP5: “Our organization’s effectiveness in delivering reliable quality products”

OP6: “Reduction in cost to reach customers”

OP7: “Reduction in overhead costs”

OP8: “Reduction in inventory costs”.

**Environmental Performance** (Gholami et al., 2013; Shahzad et al., 2020)

EP1: “Environmental performance is enhanced in terms of material reuse”

EP2: “Environmental performance is enhanced in terms of environmental compliance”

EP3: “Environmental performance is enhanced in terms of environmental preservation”

EP4: “Environmental performance is enhanced in terms of the reduction of hazardous wastes and emissions”

EP5: “Environmental performance is enhanced in terms of reduction of resource consumption (e.g., energy, water, electricity, gas, and petrol)”
